# Growth rate alterations of human colorectal cancer cells by 157 gut bacteria

**DOI:** 10.1080/19490976.2020.1799733

**Published:** 2020-09-11

**Authors:** Rahwa Taddese, Daniel R. Garza, Lilian N. Ruiter, Marien I. de Jonge, Clara Belzer, Steven Aalvink, Iris D. Nagtegaal, Bas E. Dutilh, Annemarie Boleij

**Affiliations:** a Department of Pathology, Radboud Institute for Molecular Life Sciences (RIMLS), Radboud University Medical Center, Nijmegen, The Netherlands; b Centre for Molecular and Biomolecular Informatics, Radboud University Medical Center, Nijmegen, The Netherlands; c Section Pediatric Infectious Diseases, Laboratory of Medical Immunology, Radboud Center for Infectious Diseases (RCI), Radboud Institute for Molecular Life Sciences (RIMLS), Radboud University Medical Center, Nijmegen, The Netherlands; d Laboratory of Microbiology, Wageningen University and Research, Wageningen, The Netherlands; e Theoretical Biology and Bioinformatics, Utrecht University, Utrecht, The Netherlands

**Keywords:** Colorectal cancer, cell proliferation, MTT assay, human microbiome, secretomes

## Abstract

Several bacteria in the human gut microbiome have been associated with colorectal cancer (CRC) by high-throughput screens. In some cases, molecular mechanisms have been elucidated that drive tumorigenesis, including bacterial membrane proteins or secreted molecules that interact with the human cancer cells. For most gut bacteria, however, it remains unknown if they enhance or inhibit cancer cell growth. Here, we screened bacteria-free supernatants (secretomes) and inactivated cells of over 150 cultured bacterial strains for their effects on cell growth. We observed family-level and strain-level effects that often differed between bacterial cells and secretomes, suggesting that different molecular mechanisms are at play. Secretomes of *Bacteroidaceae, Enterobacteriaceae*, and *Erysipelotrichaceae* bacteria enhanced cell growth, while most *Fusobacteriaceae* cells and secretomes inhibited growth, contrasting prior findings. In some bacteria, the presence of specific functional genes was associated with cell growth rates, including the virulence genes TcdA, TcdB in *Clostridiales* and FadA in *Fusobacteriaceae*, which both inhibited growth. *Bacteroidaceae* cells that enhanced growth were enriched for genes of the cobalamin synthesis pathway, while *Fusobacteriaceae* cells that inhibit growth were enriched for genes of the ethanolamine utilization pathway. Together, our results reveal how different gut bacteria have wide-ranging effects on cell growth, contribute a better understanding of the effects of the gut microbiome on host cells, and provide a valuable resource for identifying candidate target genes for potential microbiome-based diagnostics and treatment strategies.

## Introduction

Over the past few decades, thousands of microbial species have been identified in the human gut microbiome. Each person harbors an estimated 3.8 × 10^13^ bacteria in their guts with a relatively stable species composition.^1–3^ Changes in the composition of the gut microbiome have been linked with various diseases including colorectal cancer (CRC), the third leading cancer worldwide with an incidence that is estimated to rise in the coming decennium.^4,5^ Besides important environmental and genetic factors, the initiation and development of CRC are affected by the human gut microbiome. During tumorigenesis, the bacterial composition on the affected intestinal mucosa changes in a process that is described by the bacterial driver-passenger model.^6,7^ Bacterial drivers may facilitate the acquisition of hallmarks of cancer^8^ in mucosal cells by generating DNA-damage, reducing the epithelial barrier function, and stimulating pro-carcinogenic immune responses. As the tumor microenvironment changes in the transition from adenoma to carcinoma, bacterial passengers compete with the drivers, further shifting the microbiome toward a CRC signature.^9,10^ The newly acquired bacteria may in turn affect tumor development, by potential inhibiting, enhancing, or neutral effects on tumor cell growth.^6^ These CRC-specific changes offer the opportunity to use the bacterial community for diagnostic, prognostic, preventive, and treatment measures.^11–20^

In feces of CRC patients, the genera *Fusobacterium, Peptostreptococcus, Ruminococcus*, and *Escherichia/Shigella* are enriched, while *Bacteroides* and *Lachnospiraceae* (*Roseburia, Lachnospira, Anaerostipes*) have a lower relative abundance.^5,9,11,21–25^ Even though phyla such as *Bacteroidetes* are depleted, single species like enterotoxigenic *Bacteroides fragilis* may be enriched in some CRC patients.^25–27^ Comparing tumor tissue with adjacent normal tissue on the intestinal mucosa of CRC patients revealed that *Fusobacterium, Prevotella, Streptococcus*, and *Peptostreptococcus* were enriched in tumors while *Blautia, Escherichia, Pseudomonas*, and *Faecalibacterium* were enriched in the normal mucosa.^5,7,9,11,22-24,28^

Many different bacteria have been associated with CRC tumors,^10^ but we are only beginning to understand the different mechanisms that are involved. The effect of bacteria on cell growth may be driven by direct bacterial-to-epithelial cell-cell contact or by secreted products present in the secretome.^29,30^ Membrane-bound bacterial proteins that require cell-cell contact can activate epithelial cell signaling. For example, the passenger bacterium *Fusobacterium nucleatum* encodes the membrane protein *Fusobacterium* adhesin A (FadA) that binds to E-cadherin, activating β-catenin signaling and resulting in increased tumor growth.^31–33^ Specific *E. coli* species with the *eae* gene express the adhesin protein intimin on their membrane surface which binds to and causes lesions to gut epithelial cells, allowing bacteria to breach the colonic barrier. Once the bacteria are bound to the epithelial cells, this allows them to inhibit DNA repair proteins, further contributing to lasting DNA damage.^34–36^ Several secreted bacterial toxins are known that can bind to receptors or pass through the cell membrane into the cytoplasm. The driver bacterium enterotoxigenic *B. fragilis* secretes the metalloprotease *B. fragilis* toxin (BFT) which leads to E-cadherin cleavage and increased wnt-signaling in colon epithelial cells and to tumor formation in mice and increased cell proliferation *in vivo*.^37,38^ Bacteria such as *Escherichia coli* containing the *pks* island are able to produce a genotoxin called colibactin. Upon mucosal breach, colibactin reaches the epithelial cells and alkylates DNA, ultimately leading to tumorigenesis.^39–41^ These examples show that taxonomically diverse bacteria may lead to the acquisition of hallmark capabilities via diverse mechanisms.

The aim of this study was to examine the effects of bacterial cells and their secretomes on the growth rates of epithelial cells. We tested the effect of 157 different gut bacteria on the growth rates of five CRC cell lines and one immortalized kidney cell line. Our results revealed that different bacterial families specifically inhibit or enhance cell growth, although contrasting effects could be observed between some closely related strains. Both known virulence genes and novel microbial pathways were associated with the different growth rate changes. These results provide the first large-scale *in vitro* analysis of the effects of different microbial strains on epithelial cell growth.

## Materials and methods

### Bacterial strains

We selected 116 different gut bacteria, including species that are depleted or enriched in CRC patients whose genome sequences were available in the PATRIC database.^42^ Additionally, we selected specific bacteria without sequenced genomes (n = 39) that were strongly linked to CRC or were isolated from CRC tissue, including *Streptococcus bovis*,^43,44^ *Clostridium septicum*,^44–46^ *Clostridioides difficile*,^47,48^ *Bacteroides* sp.^27^ and the two potentially beneficial bacteria *Lactobacillus casei Shirota*^49-51^and *Akkermansia muciniphila* ATCC BAA-835^TM^.^52-55^ Together, we analyzed 157 bacterial strains isolated from the human gut microbiome (Supplementary Table S1).

We purchased 96 bacterial strains from the reference catalog of the Human Microbiome Project (HMP, Prof. Dr. Emma Allen-Vercoe from the University of Guelph, Canada); 24 bacteria were kindly provided by Prof. Dr. Cynthia L. Sears from Johns Hopkins Medical Institutions, Baltimore, MD, USA; five strains were purchased from DSMZ (*Clostridium septicum* (Macé 1889) Ford 1927 DSM7534,^56^ *C. difficile* (Hall and O’Toole 1935) Lawson et al. 2016 DSM27543 (known as *Clostridium difficile* 630^57^), *Fusobacterium nucleatum* Knorr 1922 DSM15643, *Fusobacterium nucleatum* subsp. *polymorphum* (ex Knorr 1922) Dzink et al. 1990 DSM20482, and *Peptostreptococcus stomatis* Downes and Wade 2006 DSM17678); one strain from ATCC (*Streptococcus agalactiae* ATCC13813); and 31 bacteria were in stock at the Radboud University Medical Center in Nijmegen, The Netherlands.^58–60^ Information about the bacterial strains, their origin, growth media, and their genome sequence is provided in Supplementary Table S1.

Bacteria were cultured in their respective media under anaerobic conditions at 37°C aired with nitrogen gas (N_2_, see Supplementary Table S1). Alternatively, *Ralstonia* sp. 5_2_56FAA, *Ralstonia* sp. 5_7_47FAA and *Pseudomonas* sp. 2_1_26 were grown aerobically at 37°C, and *Bacillus smithii* was grown anaerobically at its preferred temperature of 50°C. Eight media were prepared to culture bacteria (Supplementary Table S1): (i) brain heart infusion-supplemented (BHI-S, ATCC Medium 1293), (ii) BHI-S supplemented with 0.1% Tween 80 at pH 8.0 (BHI-T),^61^ (iii) NADC – 99X medium (ATCC Medium 1804), (iv) Desulfovibrio medium (ATCC Medium 2755), (v) Modified Reinforced Clostridial Medium (RCM, ATCC Medium 2107), (vi) Reinforced Clostridial medium with sodium lactate (60% solution) at a concentration of 1.5% (RCM+, from ATCC Medium 1252), (vii) differential RCM (DRCM), and (viii) nutrient broth (NB, ATCC Medium 3). Bacteria were grown until the medium was turbid and the optical density (OD) was at least 1.0. Absorbance was obtained by measuring OD at 600 nm.

### Obtaining secretomes and bacterial cells

To investigate the effect of secreted molecules, we obtained secretomes from 154 bacterial cultures as follows. After culturing, bacteria were centrifuged at 4,700 rpm for 10 min and some bacteria subsequently at a higher speed to settle them down (see Supplementary Table S1). Next, supernatants were spun at 4,700 rpm for 10 min to pellet any remaining bacteria and filter-sterilized using 0.2 µm filters (Sigma-Aldrich, USA). Molecules larger than 10 kDa were concentrated using amicon ultra-15 centrifugal filters (Merck Millipore, Merck, USA). Concentrated supernatants were frozen at −80°C until further use and are further referred to as secretomes.

To investigate the effect of bacterial surface-bound molecules, 145 bacteria were inactivated to allow cellular growth rates to be assessed by measuring metabolic activity (see the section “MTT assay” below). Cells of 12 spore-forming bacteria under our experimental conditions were excluded from this analysis (see Supplementary Table S1). Pelleted bacteria were resuspended in 70% ethanol and incubated for 5 min according to our inactivation protocol.^62^ After centrifugation at 20,000 rcf for 30 s, bacteria were washed in PBS twice and inactivated bacteria were frozen at −80°C until further use.

### Culturing of cell lines and identification of their mutational landscapes

Five CRC cell lines (Caco-2, HCT15, HCT116, HT29, and SW480) and one immortalized embryonic kidney cell line (HEK293T) were selected based on their differences in mutational landscapes.^63^ Most commonly mutated oncogenes and tumor suppressor genes in CRC were confirmed with targeted single molecule molecular inversion probe (smMIP) mutation analysis as described,^64^ with additional smMIPs targeting CXCR4 (NM_001008540.1) codons 281–357, EZH2 (NM_00004456.4) codons 471–502, 618–645, 679–704, and SF3B1 (NM_012433.2) codons 603–471, 833–906 (smMIP sequences are available upon request), at the Radboud Diagnostics Department, Nijmegen, The Netherlands (Supplementary Table S2). Cells were split twice a week using Dulbecco’s Modified Eagle Medium (DMEM, Lonza, Switzerland) supplemented with 10% fetal bovine serum (FBS, Thermo Fisher Scientific, USA) and 1% Penicillin/Streptomycin (Pen/Strep, Thermo Fisher Scientific, USA). Cells used for this study were not split for more than 25 passages.

For MTT assays, optimal cell seeding in 96 well plates (Greiner Bio-One, Austria) was defined at 5,000 cells/well for Caco-2, 4,000 cells/well for HCT15, 1,500 cells/well for HCT116, 10,000 cells/well for HT29, 6,000 cells/well for SW480, and 20,000 cells/well for HEK293T. Cells were incubated overnight to attach before the addition of secretomes or bacterial cells.

### MTT assay

Secretomes were diluted to their original concentrations in DMEM supplemented with 10% FBS and 1% Pen/Strep. Inactivated bacteria were diluted with DMEM supplemented with 10% FBS and 1% Pen/Strep to an OD of 0.1 and incubated on cell lines up to 72 h. For secretomes, filter-sterilized bacterial culture media used to generate secretomes served as a control for cell growth in the assay, while for bacterial cells, DMEM supplemented with 10% FBS and 1% Pen/Strep served as control for cell growth. MTT assays were performed to measure the cell metabolic activity every 24 h. These assays involve the conversion of the water-soluble yellow dye MTT [3-(4,5-dimethylthiazol-2-yl)-2,5-diphenyltetrazolium bromide] to the insoluble purple formazan by the action of mitochondrial reductase. At 24, 48 and 72 h 10 µl of MTT (5 mg MTT dissolved per mL PBS) dye was added to the wells and cells were incubated for 3 h. Formazan was then solubilized with 150 µL MTT solvent (0.5 mL of 10% Nonidet (dissolved in water), 50 mL isopropanol (2-propanol) and 16,7 µL hydrochloric acid fuming 37% together) and the concentration determined by optical density at 570 nm (BioRad microplate reader model 680). Metabolic activity of cell lines was used as a measure for cell growth.^65^ All experiments were performed in quadruplicate.

### Cell growth analysis

Absorbances at an optical density of 570 nm were measured by MTT assays at four time points (0, 24, 48, and 72 h). Growth rates per hour were calculated by dividing the difference in absorbance by the time interval between measurements (24 h). Results from four independent experimental replicates were averaged. Growth rates between 24 and 48 h were selected for comparative analysis because that time window captured the optimal growth for most cell lines (Supplementary Figure S1).

A growth rate score was defined by subtracting the growth of negative controls (cells cultured without bacterial products) from those cultured with bacterial products (secretomes or bacterial cells). Scores greater than 0 refer to enhanced growth, while scores lower than 0 refer to inhibited growth (see Supplementary Tables S3 and S4). To allow comparability between cell lines, normalized growth rate scores were calculated by dividing per cell line the scores (g) of each of the n bacteria by the norm (|g|) of the experimental condition $(\left|g\right|=\sqrt{\overset{n}{\underset{i=i}{\sum }}{g_{i}}^{2}}$). The normalized growth rate scores were used to evaluate whether the enhancing or inhibiting effect of a bacteria is significantly stronger than the mean effect observed for all other bacteria. For this purpose, growth rate scores were transformed into z-scores and *p*-values were computed by separate one-tailed z-tests for enhancing or inhibiting effects. Based on these *p*-values, strains were identified as enhancers or inhibitors of growth by using a cutoff of *p* < .05. This cutoff represents the 5% extremes of the distribution of z-scores and were here used to identify the strongest effects. The FDR corrected *p*-values were also reported (Supplementary Tables S3 and S4).

The overall effects of bacterial secretomes and inactivated cells were visualized by projecting their normalized growth scores in a two-dimensional space with the t-SNE algorithm.^66^ To evaluate if bacteria of the same family have a similar overall effect on cell growth relative to bacteria from other families, we applied unsupervised nearest neighbor clustering^67^ and evaluated how often bacteria from the same family were nearest neighbors compared to a random sample without replacement. For this analysis, we only used bacteria from families with more than four representatives. Random groups were evaluated per family and were defined to contain the same number of strains as the family. Fisher’s exact test was used to evaluate if the nearest neighbor groups were enriched for bacteria of the same family compared to 1,000 random groups of the same size. The harmonic means of *p*-values from this comparison are reported.

### Association of bacterial virulence genes to growth rate changes

Bacterial genomes were screened for 35 known virulence genes that may be associated with cancer or epithelial cell changes (Supplementary Table S5). The virulence factors Map (Mitochondrial associated protein), Type-3 secretion system (T3SS) effector protein of *Citrobacter rodentium*, Shiga toxin 2A (Stx2A), Shigella enterotoxin 1 (ShEt1), and colibactin (cyclomodulin toxin on polyketide synthase (pks) island of *Enterobacteriaceae*) were identified in the *Enterobacteriaceae* family, *B. fragilis* toxin (BFT) in *B. fragilis* species, and FadA in the *Fusobacteriaceae* family (Supplementary Table S5). *Clostridium difficile* toxin A, and B (TcdA/TcdB) in *Clostridioides difficile (Prévot 1938) Lawson et al. 2016*, and Alpha-toxin of *Clostridium septicum* (Mace 1889) Ford 1927 were identified as secreted factors in previous reports.^56,57,68^ Growth rate changes of strains containing virulence genes (if present in ≥2 strains) were compared to those of their non-virulent counterparts within the order (*Clostridiales*), family (*Fusobacteriaceae* and *Enterobacteriaceae*) or species (*B. fragilis)*. Groups were compared using Mann-Whitney U-test of aggregate data of all cell lines. *P* < .05 was considered significant.

### Genes and subsystems identification

To identify genes that are associated with the inhibiting/enhancing effects of bacterial strains within families, we sorted all strains based on their effect on growth rate scores and used a normalized Kolmogorov–Smirnov statistic to quantify the association of a gene with the observed effect. The significance of the rank-based statistic was estimated by comparing its value to values obtained from 10^3^ random permutations of the same list. This provided all genes encoded by the genomes from a family with a *p*-value which quantified the association of the gene to the inhibiting/enhancing effect. We evaluated if genes that belong to the same functional subsystems^42,69^ were more often found to be significantly associated with the inhibiting/enhancing effect than to 10^3^ random groups of genes. We defined this association by modeling the average count of genes belonging to a functional subsystem within the random groups as Poisson distributed variables. We then tested if the count of genes with significant *p*-values was statistically higher than expected from the Poisson model, with a significance level of 0.05 after Benjamini/Hochberg correction for multiple tests. We used the Poisson distribution as the null distribution since (i) the labels for functional subsystem within the random groups are independent; (ii) Counts are expressed as averages from many random samples; (iii) the mean and variance are equivalent, all features consistent with a Poisson distribution.^70^

## Results

### Reproducibility of growth rate alterations by bacterial cells and secretomes

To identify alterations in the growth rates of CRC cell lines by bacterial cells or their secreted products, inactivated cells or secretomes were added to five CRC cell lines with different mutational landscapes and one embryonic kidney cell line HEK293T (see Methods and Supplementary Table S2). HEK293T cells are immortalized by DNA of human adenovirus type 5 resulting in senescence of the pRb and p53 pathway.^71^ While these cells are not normal, they have no mutations in the genes that are commonly mutated in CRC, as confirmed by our smMIP analysis (Supplemental Table 2). For each cell line independently, plain cell culture and bacterial culture media served as a control for cell growth for cells and secretomes, respectively. A total of 7,176 experiments were conducted where we measured the bacterial effect on cell growth. Each experiment was repeated four times. We evaluated the reproducibility of these replicates by performing linear regression over all pairwise combinations of replicates, i.e. measurements for all the possible combinations of two replicates for every strain appended into two separate lists. As shown in Supplementary Figure S2, we found high reproducibility between replicates (regression coefficient: 0.87 ± 0.05, intercept: 0.04 ± 0.02). Based on this reproducibility, we averaged the four experimental replicates for further analysis.

### Contrasting effects between bacterial cells and secretomes

**Figure 1.**
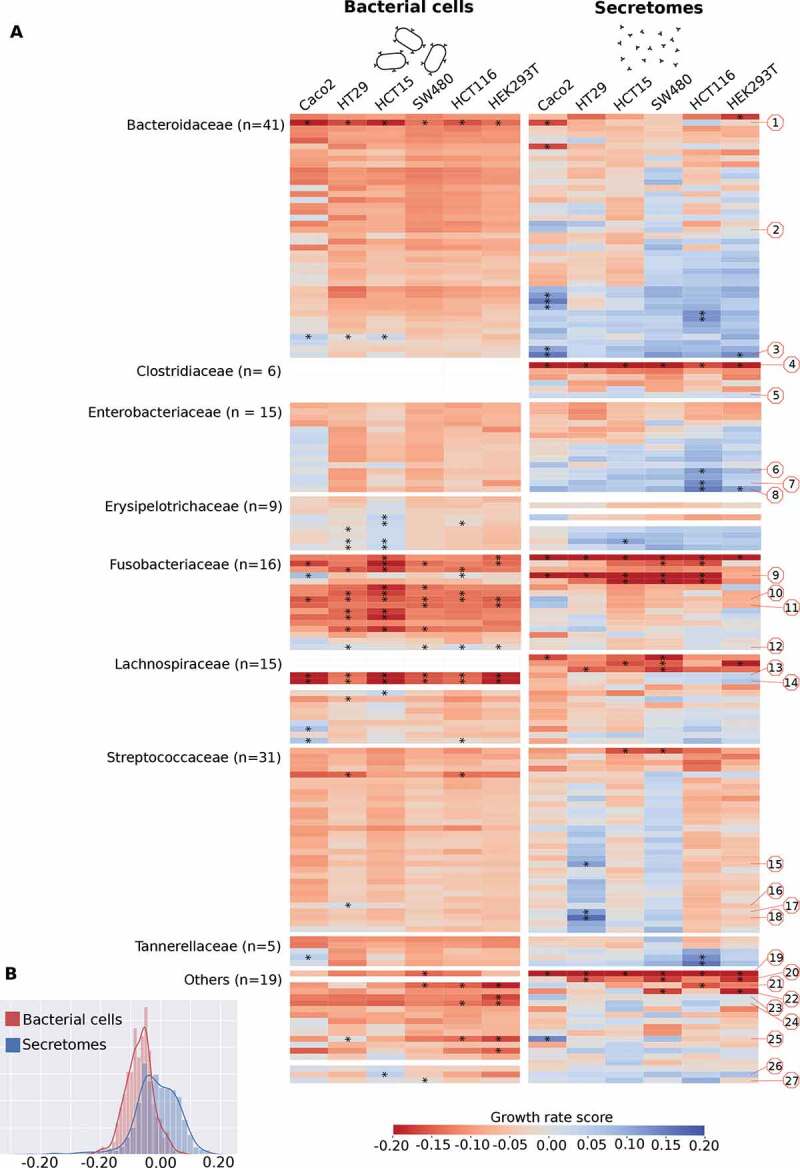
Growth rate scores of six human cell lines upon treatment with bacterial cells and secretomes. (a) Heatmap indicating low and high growth rate scores, respectively red and blue. Bacteria are sorted within bacterial families by the average growth rate score. Red numbered octagons highlight the strains discussed in the text: *Bacteroides sp*. 2_1_22 (1), *B. fragilis* K570 clinda R (ETBF) (2), *B*. sp. 4_1_36 (3), *Clostridium septicum* (Mace 1889) Ford 1927 (4), *C*. sp. D5 (5), *Escherichia coli* D9 (6), *Klebsiella* sp. 1_1_55 (7), *E. coli* 4_1_47FAA (8), *F. nucleatum* DSM 15643 (ATCC 25586) (9), *F. nucleatum* DSM 20482 (ATCC 10953) (10), *F. necrophorum subsp. funduliforme* 1_1_36S (11), *F. nucleatum subsp. animalis* 11_3_2 (12), *Lachnospiraceae bacterium* 8_1_57FAA (13), *L. bacterium* 3_1_46FAA (14), *Streptococcus bovis* 1212 (15), *S. bovis* 1459 (16), *S. bovis* 1417 (17), *S. bovis* 207 (18), *Pediococcus acidilactici* 7_4 (19), *Pseudomonas* sp. 2_1_26 (20), *D*. sp. 6_1_46AFAA (21), *Ralstonia* sp. 5_2_56FAA (22), *Ruminococcaceae bacterium* D16 (23), *Synergistes* sp. 3_1_syn1 (24), *Desulfovibrio sp*. 3_1_syn3 (25), *Propionibacterium sp*. 5_U_42AFAA (26), and *Eubacterium sp*. 3_1_31 (27). Highlighted with asterisks are cases that correspond to the 5% extremes of the z-score distribution. (b) Distribution of the growth rate scores for bacterial cells and secretomes. Histograms show the distribution of the values from the two heatmaps above, indicating that on average bacterial cells exhibit a stronger inhibiting effect when compared to secretomes.

Figure 1(a) summarizes the effect of bacterial cells and secretomes on the growth of human cell lines. Overall, bacterial cells exhibit a stronger inhibiting effect than secretomes (*p* = 1.02e-75, Wilcoxon signed rank test, Figure 1(b)). There was a low correlation between the overall effects of secretomes and inactivated bacteria on cell growth (Pearson r = 0.176). A low correlation was also observed when the measurements were stratified per bacterial family (Supplementary Table S6), indicating that specific factors within the two compartments (cell wall attached or secreted molecules) are of importance for the observed effects.

### Difference in growth rate alterations between CRC cell lines

**Figure 2.**
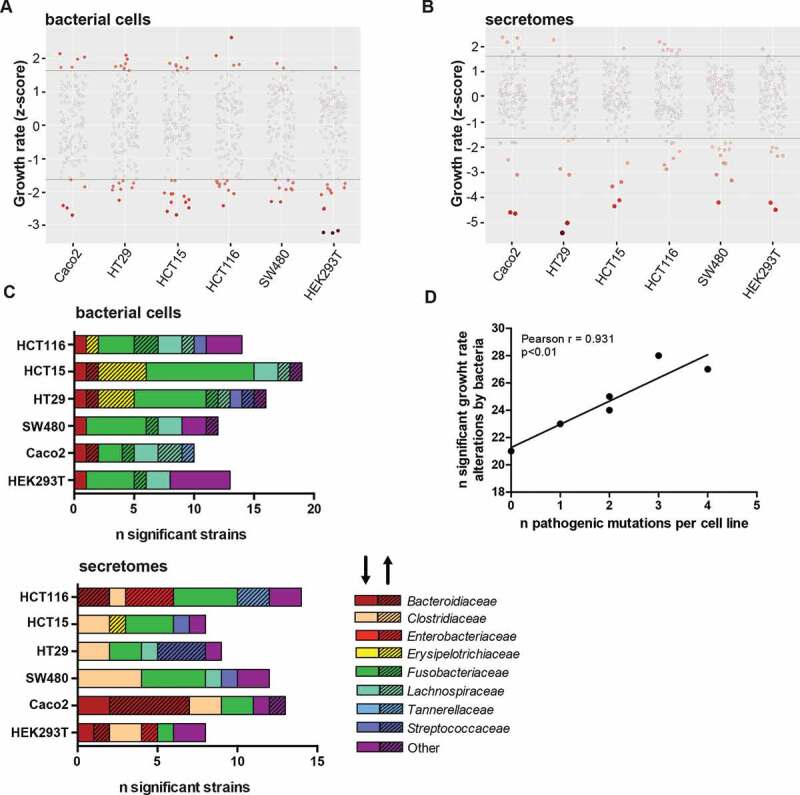
Growth rate alterations of cells lines upon incubation with bacterial cells and secretomes. Distribution plots illustrating effects of (a) bacterial cells and (b) secretomes on cell lines. Negative numbers indicate growth inhibition whereas positive numbers show growth enhancement. The color intensity of the circles represents significance of the effect on growth. (c) Overview of the number of bacterial cells and secretomes significantly enhancing or inhibiting cell growth per tested cell line, sorted by bacterial family. The shading of the bar (arrow pointing upwards) highlights enhancing and unshaded bar (arrow pointing downwards) highlights inhibiting strains. (d) Correlation of number of strains significantly altering growth rates to the number of pathogenic mutations present in cell lines. HEK293T, Caco-2, HT29, SW480, HCT116, and HCT15 were shown to possess 0, 1, 2, 2, 3, and 4 pathogenic mutations, respectively.

Growth rates were significantly enhanced or inhibited (*p* < .05) in one or more cell lines in 35 out of 145 (24.1%) inactivated bacterial strains (Figure 2(a), Supplementary Table S3) and 33 out of 154 (21.4%) secretomes (Figure 2(b), Supplementary Table S4). Growth rates of HEK293T cells were affected by the lowest number of bacteria (n = 21, n = 3 enhanced and n = 18 inhibited growth). On the other end of the spectrum were HCT116 cells that were affected by 28 different bacteria (n = 11 enhanced and n = 17 inhibited growth) and HCT15 cells that were affected by 27 different bacteria (n = 8 enhanced and n = 19 inhibited; Figure 2(c)). These two cell lines had microsatellite instability (MSI) and harbored the greatest number of pathogenic mutations in known tumor suppressor and oncogenes (n = 3 and n = 4, respectively, see Supplementary Table S2). In general, the number of pathogenic mutations of a cell line correlated with the number of bacteria that significantly affected its growth (Pearson r = 0.931, *p* < .01, see Figure 2(d)), suggesting that acquiring hallmark mutations might make cells more susceptible to growth rate changes by bacteria.

Some strains significantly enhanced or inhibited the growth rate of specific cell lines (Figure 1). *Streptococcaceae* cells generally had a neutral effect on cell growth, however, some *Streptococcaceae* secretomes specifically enhanced the growth of HT29 and SW480 cells (Figure 1). Three *Streptococcus bovis* secretomes (strains 207, 1212 and 1417) and one inactivated bacterium (strain 1459) selectively enhanced growth in HT29 cells, while no effects were observed in other cell lines (Figures 1 and 2(c)). The genomes of these enhancing *Streptococcus bovis* strains cluster together in a phylogenetic tree with *Streptococcus gallolyticus* subsp. *gallolyticus* (SGG) and subsp. *pasteurianus* (SGP) (Supplementary Figure 3). Other notable cell-line specific effects include *Erysipelotrichaceae* cells, and *Tannerellaceae* secretomes that significantly enhance the growth of HCT15 and HCT116 cells, respectively.

### Bacterial family-level and strain-level effects on cell growth rates

**Figure 3.**
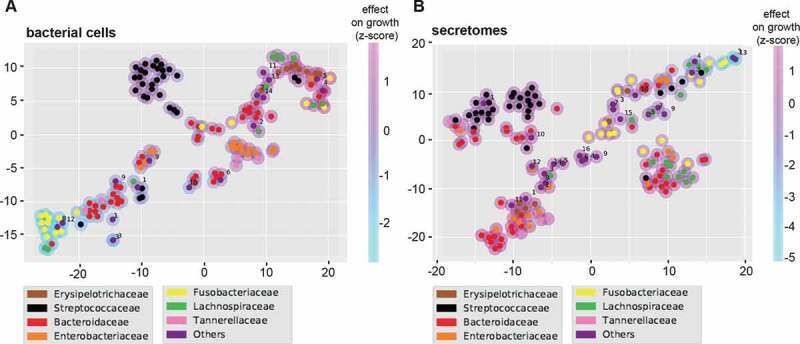
T-SNE plots summarizing the overall growth effects of bacterial cells (a) and secretomes (b) on six human cell lines. Colored circles correspond to strains belonging to particular bacterial families. Magenta and light blue shading around the circles indicate enhancing and inhibiting effects of strains, respectively. Families labeled as “Others” contains the following strains for bacterial cells (A): *Lactobacillaceae* (1), *Peptostreptococcaceae* (2), *Desulfovibrionaceae* (3), *Eubacteriaceae* (4), *Bifidobacteriaceae* (5), *Enterococcaceae* (6), *Veillonellaceae* (7), *Synergistaceae* (8), *Burkholderiaceae* (9), *Pseudomonadaceae* (10), *Propionibacteriaceae* (11), *Ruminococcaceae* (12), *Akkermansiaceae* (13), *Acidaminococcaceae* (14). And the following strains for secretomes (B): *Lactobacillaceae* (1), *Eubacteriaceae* (2), *Clostridiaceae* (3), *Peptostreptococcaceae* (4), *Bifidobacteriaceae* (5), *Enterococcaceae* (6), *Veillonellaceae* (7), *Synergistaceae* (8), *Burkholderiaceae* (9), *Desulfovibrionaceae* (10), *Propionibacteriaceae* (11), *Ruminococcaceae* (12), *Pseudomonadaceae* (13), *Akkermansiaceae* (14), *Acidaminococcaceae* (15), *Bacillaceae* (16).

To assess the overall effect of the different bacterial secretomes and cells on our six cell lines, we projected the growth rate scores into a two-dimensional t-SNE plot. Figure 3 shows this lower dimensional space overlayed with family-specific colors and a shade corresponding to the average growth effect. We observed a clear clustering of most, but not all of the bacterial families, i.e. cells or secretomes from the same families tend to have similar effects on the different cell lines (see also Figure 1 and Table 1). To confirm this observation, we tested if the growth effects of bacteria from the same family were more similar than one would expect by chance by permuting the data labels 1,000 times (see Methods). The effect of bacterial cells and secretomes was found to be significantly clustered for five and three bacterial families, respectively (Supplementary Table S7). For bacterial cells, the most striking effect was observed for *Fusobacteriaceae* that generally inhibited cell growth. Most *Bacteroidaceae, Enterobacteriaceae*, and *Erysipelotrichaceae* secretomes enhanced cell growth, although not always significantly (Figure 1). Contrastingly, *Clostridiaceae* secretomes generally inhibited growth rates.

**Table 1.** Secretomes and bacteria significantly enhancing or reducing cell growth rate (z-scores).




Secretomes are highlighted in gray, green represents significant enhancement (*p* < 0.05) in cell growth rate and red represents significant reduction (*p* < 0.05) in cell growth rate.

In the *Bacteroidaceae* family (n = 41 strains tested), secretomes and bacterial cells of seven and one strains significantly enhanced cell growth, respectively. The effects were most prominently observed in Caco-2 cells, while the growth of HEK293T cells was only significantly enhanced by the secretome of *Bacteroides* sp. 4_1_36 (Figure 2(c) and Table 1). Only three *Enterobacteriaceae* secretomes (n = 15) significantly enhanced the growth of HEK293T cells (*Escherichia coli* 4_1_47FAA) and HCT116 (*Escherichia coli* 4_1_47FAA and D9, and *Klebsiella* sp. 1_1_55) while no effects were observed for bacterial cells.

Growth-inhibiting effects were mainly observed within the family *Fusobacteriaceae* (Figure 1 and Table 1). From the 12 out of 16 significant *Fusobacteriaceae* (81.3%) cells, 2 enhanced and 10 inhibited cellular growth. *Fusobacterium necrophorum* subsp. *funduliforme* 1_1_36S cells enhanced growth in HCT116, SW480, and HEK293T cells. Strikingly, while *Fusobacterium nucleatum* subsp. *animalis* 11_3_2 cells enhanced the growth of Caco-2 and HCT116, its secretome inhibited growth in those same cell lines. Especially the cell line HCT15 was sensitive to *Fusobacteriaceae* growth inhibition where 11 out of 16 strains significantly reduced growth rates. Alternatively, HEK293T and Caco-2 were less sensitive to *Fusobacteriaceae* with only four out of 16 strains inhibiting cell growth. Notably, the effect of *Fusobacteriaceae* secretomes on cell growth may be cell-specific depending on, for example, mutation status, since most secretomes that inhibited HCT116, SW480 and HCT15, did not inhibit the growth of Caco-2 and HEK293 T cells.

### Families with fewer than 5 strains tested

Among strains from families with less than five tested strains (n = 18), *Eubacterium sp*. 3_1_31 and *Propionibacterium sp*. 5_U_42AFAA cells increased cell growth rates in SW480 and HCT15 cells, respectively (Table 1). Interestingly, *Pseudomonas* sp. 2_1_26 secretomes significantly inhibited the growth rates of all cell lines while the cells specifically inhibited SW480. *Pediococcus acidilactici* 7_4 and *Ruminococcaceae bacterium* D16 cells selectively inhibited HEK293T, while *Synergistes sp*. 3_1_syn1 also inhibited HCT116. Aerobically cultured *Ralstonia* sp. 5_2_56FAA secretomes inhibited 2 cell lines, SW480 and HEK293T. For *Desulfovibrio* sp. 3_1_syn3, the secretome increased Caco-2 growth while the bacterial cells increased HT29 growth. Contrastingly, these same bacterial cells decreased the growth of HCT116 and HEK293T. Similarly, *Desulfovibrio* sp. 6_1_46AFAA cells decreased the growth rates of HCT116, SW480 and HEK293T cells.

### Strain-specific effects that contrast their family

While strains from the same bacterial families tend to have similar effects on the growth rates of cell lines (Supplementary Table S7), large variations were observed within certain families. For instance, within the *Clostridiaceae* family, the secretome of *Clostridium septicum* (Mace 1889) Ford 1927 strongly inhibited the growth of all tested cell lines, while the *Clostridium* sp. D5 secretome enhanced growth, although the significance threshold was not reached (Figure 1 and Supplementary Table S4). Similarly, *Bacteroides* sp. 2_1_22 cells strongly inhibited the growth rates of all cell lines while *B. fragilis* K570 cells enhanced growth in most cell lines (Figure 1 and Supplementary Table S3). A large growth rate variation was also observed in cell lines exposed to *Lachnospiraceae* cells (n = 11). For this family, the cells of *L. bacterium* 3_1_46FAA and *L. bacterium* 8_1_57FAA significantly inhibited the growth rates of all cell lines, while most of the other strains showed weak or low inhibition.

The observed variations in growth effect within bacterial families might either be explained by the phylogenetic distances between the strains or by characteristics with a non-phylogenetic distribution, such as accessory functions. To evaluate this, we measured the distances on a phylogenomic tree and compared them with the differences in growth rate effects over the cell lines. For most bacterial families, there is no association between the growth rate effects and phylogenetic distances (Supplementary Table S8, Supplementary Figure S4), suggesting that these effects may be associated with accessory functions that are independently gained or lost in different lineages. For *Erysipelotrichaceae* secretomes and cells, *Fusobacteriaceae* cells, and *Lachnospiraceae* secretomes, we found that the phylogenetic distance was significantly correlated with the growth rate effects, suggesting that these effects may be associated to variations in universally shared genes or to accessory functions with phylogenetically consistent dynamics.

### The presence of virulence genes is associated with growth rate inhibition

To explain the complex, sometimes strain-dependent patterns of growth enhancement or inhibition, we analyzed the genome sequences of 144/157 tested gut bacteria. First, we checked whether 35 known bacterial family-specific virulence genes were present within the genomes of the tested strains (Supplementary Table S9), because some of the encoded virulence factors have been linked to cell proliferation changes. Nine toxin genes were found, including TcdA, TcdB,^72^ alpha-toxin^68^ of *Clostridiales*; FadA;^31^ colibactin present on the pks-island;^73,74^ Shiga toxin ShEt1; *Shigella* enterotoxin Stx2A; effector protein Map secreted by T3SS of *Citrobacter rodentium* and *Escherichia coli*;^75,76^ and BFT.^38^

**Figure 4.**
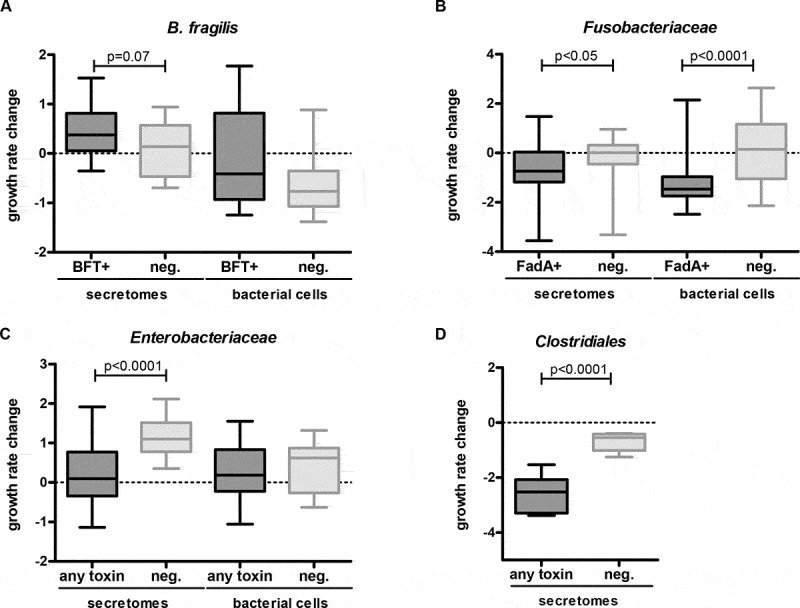
Influence of strains with and without encoded virulence factors on cellular growth rates. *B. fragilis* (a), *Fusobacteriaceae* (b), *Enterobacteriaceae* (c) and *Clostridiales* (d) encoding the virulence factors *bft, fadA*, any toxin (*Map, pks, ShEt1, Stx2A*), and any toxin (*TcdA, TcdB, alpha-toxin)*, respectively, were compared to strains without these encoded virulence factors (Mann-Whitney U test). A trend toward cell growth enhancement was observed for *B. fragilis bft*+ secretomes (*p* = .07) (A), while significant inhibition of cell growth was observed for *fadA*+ *Fusobacteriaceae* cells (*p* < .0001), and secretomes (*p* < .05) (B), for *Enterobacteriaceae* secretomes encoding toxins (*p* < .0001) (C), and for *Clostridiales* secretomes encoding toxins (*p* < .0001) (D).

As shown in Figure 4, strains containing virulence genes tend to change growth rates, relative to related strains without those virulence genes. For example, the secretomes of *Clostridiales* strains significantly inhibited growth rate if their genomes contained *TcdA, TcdB* or *alpha-toxin* (Figure 4(a), *p* < .0001). *TcdA* and *TcdB* secretion by *Clostridioides difficile (Prévot 1938) Lawson et al. 2016* was confirmed by ELISA (data not shown). Similarly, *Fusobacteria* strains encoding the membrane protein *FadA*, which might be responsible for growth rate changes of epithelial cells,^31^ inhibited growth rates significantly (Figure 4(b), *p* < .0001 and *p* < .05 for bacterial cells and secretomes, respectively). Within the *Enterobacteriaceae* family, multiple secreted toxins were identified in the genomes, including colibactin, *ShEt1, Stx2A*, and *Map*. Secretomes of *Enterobacteriaceae* possessing any of these toxins inhibited cell growth relative to strains without these toxins (Figure 4(c), *p* < .0001). No differences were observed between bacterial cells of *Enterobacteriaceae* with or without the toxin genes. The secretomes of three *B. fragilis* strains encoding the secreted enterotoxin *bft* enhanced the growth rates of CRC cells, while the five *B. fragilis* strains without the toxin did not, although this trend was not significant (Figure 4(a), *p* = .075). The trend was not observed for bacterial cells, where only *B. fragilis* K570 significantly enhanced growth rates (Table 1).

### Functional categories associated with differential effects within families

Besides the toxin genes described above, other genes might also play a role in the observed effects. Thus, we next performed an unbiased comparative genome analysis where we associated all annotated bacterial genes to the effect of the strain on cell growth. We next grouped the genes by functional category and assessed whether genes within a category were significantly associated with cell growth alterations. Based on the PATRIC^42^ annotations we evaluated genes in two hierarchical levels of functions, namely ‘Subsystems’ and ‘Superclass’. All functional categories found to be significantly associated with cell growth alterations are listed in Supplementary Table S10.

The “Membrane transport” superclass was significantly associated with inhibiting *Enterobacteriaceae* cells and secretomes, and with enhancing *Fusobacteriaceae* secretomes. Other enriched superclasses included “Cell envelope”, “Metabolism”, “Cellular processes”, “Protein processing”, “Energy”, and “Stress response, defense and virulence” (Supplementary Table S10). Subsystems including “Cobalamin synthesis”, “Ethanolamine utilization”, and the “Dpp dipeptide ABC transport system” were consistently found to be associated with growth enhancement or inhibition in multiple bacterial families and in multiple cell lines (Supplementary Table S10). The significant effects of other subsystems including “Vir-like type 4 secretion system”, “Glutamate fermentation”, “Flagellum”, “Branched-chain amino acid biosynthesis”, and others were associated with specific bacterial families. We expect that these statistical associations will provide valuable leads for future experiments.

## Discussion

Over 150 human gut bacteria belonging to taxonomic groups that have previously been associated with CRC were tested for their effects on cell growth of five CRC cell lines (HT29, Caco-2, HCT116, SW480, and HCT15) with different mutation landscapes representing commonly mutated genes in CRC, and one immortalized kidney cell line (HEK293T). Our results show that cell growth enhancing and inhibiting effects of gut bacteria depend both on the mutational landscape of the cells and on the identity and functional content of the bacteria, including secreted or cell wall attached factors. CRC is a heterogeneous disease, as is reflected in the five different CRC cell lines with mutations in key CRC related genes (*KRAS, APC, BRAF, TP53, CTNNB1*, and *PIK3CA*). These mutations are absent from the HEK293T cells, an immortalized kidney cell line that was transformed by DNA of a human adenovirus, causing senescence in the pRb and p53 pathway.^71^ HEK293T cells were affected by the lowest number of gut bacterial strains, with only small differences.

### Closely related bacteria may have distinct effects on cell growth

A striking observation was that the effects of bacterial cells and secretomes were strongly associated with bacterial families, although exceptions to this general pattern were observed. Previous studies have found that *Fusobacteria, Streptococcus, Peptostreptococcus, Ruminococcus*, and *Escherichia/Shigella* tend to be enriched in tumors, while *Bacteroides, Pseudomonas, Lachnospira*, and *Anaerostipes* are depleted.^5,7,9,22-24,28,31,41,77-80^ Regardless of the enrichment or depletion on CRC tumors *in vivo*, our *in vitro* results indicate that closely related bacteria may have distinct effects on cell growth. For example, we observed that different *Fusobacteria* strains, which were all cultured in the same media under the same conditions, may have opposing effects on cell growth, while these bacteria are consistently enriched in patients.^24,28,81^ This suggests that specific bacterial factors may play a role.

### Fusobacteriaceae: enhancing or inhibiting growth?

Previous studies indicated that *Fusobacteriaceae* enhanced the growth of HCT116, SW480, and HT29 cells.^31,80^ *F. nucleatum* ATCC25586 was shown to enhance CRC cell growth via interaction with Toll-like receptors TLR4, TLR2, and myeloid differentiation primary response protein 88 (MYD88),^80^ and the *F. nucleatum* strain Fn12230 was previously shown to enhance cell growth through the cell-wall attached adhesin FadA.^31^ Our results showed that most *Fusobacteriaceae* significantly inhibited cellular growth, including *Fusobacterium nucleatum* ATCC25586, contrasting previous observations,^72,80^ and that the presence of the *FadA* gene was significantly linked to growth inhibition by cells and secretomes (Table 1). Notably, the secretomes showed less pronounced inhibition than bacterial cells. While FadA is membrane-bound, secretomes likely include outer membrane vesicles (OMVs) that are 40–110 nm in size and express FadA.^32,82^ To rule out the possibility that these contrasting results were due to differences in methods, we compared our high-throughput MTT-based assay, used in this study, with the cell counting assay, that was employed in the referred studies, on six cell lines, using six different secretomes, and found a high correlation (Pearson’s r = 0.813, Supplementary Figure S5). These results confirm that the observed inhibitory effects by *Fusobacteriaceae* strains do not depend on the applied experimental techniques, but may partly be due to the use of different *Fusobacteriaceae* strains (Fn12230, ATCC25586, ATCC23726) isolated from extra-intestinal sites.^83^ Specifically, HCT15 cell growth was significantly inhibited by 11/16 *Fusobacteriaceae* strains (nine cells and three secretomes). HCT15 cells contain a missense mutation in MYD88 (p.Arg264Ter) resulting in an MYD88 deficiency, and may thus explain the insensitivity of this cell line to *F. nucleatum* subsp. *animalis* 11_3_2 and *F. necrophorum* subsp. *funduliforme* 1_1_36S, the two *Fusobacteriaceae* strains that promoted the growth of the other cell lines, but not of HCT15.

### Virulence factors are associated to cell growth inhibition

The growth-inhibiting properties of *Clostridiaceae* secretomes may result from toxins encoded by this family. We observed that *Clostridioides difficile (Prévot 1938) Lawson et al. 2016* and *Clostridium septicum* (Mace 1889) Ford 1927 secreting the virulence factors TcdA and TcdB, and lethal alpha toxin, respectively, significantly inhibited cell growth compared to the other *Clostridiales* secretomes. *Clostridium septicum* has been associated with CRC in the past.^14,15^

*Enterobacteriaceae* strains encoding the toxins colibactin, *Stx2A, ShEt1*, and *Map* inhibited cell growth relative to non-toxigenic *Enterobacteriaceae* strains. Colibactin (present in four of our *Escherichia* strains) is a genotoxic compound that alkylates DNA and generates DNA double-strand breaks.^41,78^ The DNA damaging effect of colibactin may not directly affect cell growth in our experimental setup, although colibactin may induce cell cycle arrest via SUMOylation of TP53 causing inactivation.^73,74^ Cell cycle arrest would stall cell growth, which can only happen in *TP53* wild-type cell lines with active TP53 protein. Indeed, the only cell lines that were relatively inhibited by strains encoding colibactin were the wildtype *TP53* cell lines HEK293T and HCT116.

An important virulence factor of *B. fragilis* is BFT.^84^ While in previous research, BFT was shown to enhance cell growth of HT29/c1 cells via β-catenin nuclear signaling,^38^ enterotoxigenic *B. fragilis* secretomes (VPI13784 (*bft1*), 86–5443-2-2 (*bft2*), and K570 (*bft3*)) only marginally enhanced cell growth in our assay compared to the other *B. fragilis* secretomes in our screen. The mild effects as observed may depend on the level of toxin production and secretion under our experimental conditions. Interestingly, enterotoxigenic *B. fragilis* str. Korea 570 cells significantly enhanced growth, which might be independent of BFT and related to other cell-wall-attached factors.

### Streptococcus bovis strains specifically enhance growth of HT29 cells

*Streptococcus bovis* infections have been linked to colorectal neoplasia.^43,85,86^ Our results demonstrate that secretomes of *Streptococcus bovis* strains that are closely related to known *S. gallolyticus* and *S. pasteurianus* strains (Supplementary Figure S3), selectively enhance the growth of HT29 cells. This is in line with a previous study showing cell growth enhancing effects in HT29 and HCT116 cells that were mediated through *β-*catenin, while no effect was observed in SW480 and HEK293T cells.^87^ An intact *β-*catenin pathway may, therefore, be required to observe the specific effect of *S. gallolyticus* on cell growth as is the case in HT29 cells,^88^ which may thus be sensitive to the effects of *S. gallolyticus* in our assay. HCT116 cells have a mutation in *CTNNB1 (p.Ser45del)* that interferes with *β-*catenin degradation, which may explain its insensitivity to *S. gallolyticus*. Alternatively, mutations in the *APC*-gene in the other CRC-cells^89^ may interfere with *β-*catenin signaling depending on the specific mutation.

### Bacterial functions associated with cell growth alterations

Several functional categories were significantly associated with cell growth. Most of these functions were identified in specific bacterial families and consistently inhibited or enhanced cell growth in different cell lines. These functions may reflect novel pathways of bacterial interference with cell growth which, to our knowledge, have not been previously identified. Most functions are related to cell metabolism, secretion, and transport systems. For example, secretomes of *Enterobacteriaceae* that encode the “Vir-like type 4 secretion system” inhibited the growth of all cell lines. Similarly, the gene superclass “Membrane transport” was mostly associated with secretomes that inhibited cell growth (Supplementary Table S10). Molecules that are secreted by these bacterial transport systems may be responsible for the inhibiting effect. For example, the Vir-like type 4 secretion system allows bacteria to secrete proteinaceous effectors that kill competitors.^90^ Here, we report an important first step in understanding the cell growth enhancing or inhibiting effects of human gut bacteria by identifying putative transport systems for effector molecules.

While the growth effects of secretomes were mostly associated with membrane transport functions, effects on cell growth by bacterial cells were associated with metabolic pathways (Supplementary Table S10). It is possible that these associations may be attributed to metabolic enzymes that may still be, although the bacterial cells were inactivated. In other ecosystems, metabolic enzymes of dead bacteria have been shown to remain active for up to 96 h.^91^ Our experiment was performed within 72 h. The synthesis of cobalamin (vitamin B12), which is important for human cell proliferation *in vitro*^92^ and a common addition to cell culturing media, was significantly associated with the cell growth enhancing effect of *Bacteroidaceae* and *Enterobacteriaceae* cells. Several possible scenarios can be envisioned to explain this observation. First, it remains to be tested whether vitamin B12 was retained in the bacterial fraction after washing, stimulating human cell growth on those cells. Second, different human gut-associated *Bacteroides* strains contain surface-exposed lipoproteins with high affinity for cobalamin.^93^ Such molecules may remove cobalamin directly from the media, making it unavailable to the human cells and impeding their growth.

The “Ethanolamine utilization” pathway was another metabolic process that we found to be associated with the inhibiting effect of *Fusobacteriaceae* cells on the growth rate of CRC cell lines. Ethanolamine (EA) is the basal component of phosphatidylethanolamine, a major phospholipid in animal cell membranes and is utilized by bacteria including *Fusobacteria* as a carbon source in the gut.^94–96^ EA sensing regulators have been proposed to regulate virulence in *Enterobacteriaceae*.^96,97^ It remains to be tested if the EA in human cell membranes triggers virulence factors in *Fusobacteria* that encode EA utilization genes, or perhaps if inactivated *Fusobacteria* cells retain the capacity to digest and kill human cells by the activity of EA utilization enzymes.

Overall, the statistical associations found in this exploratory analysis provide valuable clues about the possible functions and mechanisms that drive the interactions of bacteria with human cells in the gut. Future experiments are needed to confirm whether these factors are causal and to further clarify the molecular mechanisms involved.

### Outlook

Our broad screen revealed many significant associations between gut bacteria, their encoded functions, and growth enhancement or inhibition of human cell lines. Many growth rate effects remain elusive and need to be further examined, such as their specificity for CRC. Moreover, it will be important to investigate the growth rate effects of living bacterial communities or mock communities, which are thought to synergize in affecting tumorigenesis,^53^ but fall outside the scope of our present study. Bacterial effects should be examined in more natural environments that mimic the conditions in the human gut, e.g. in co-culture with mucus, immune cells, and/or within organoid cultures.^98^ Our study forms an important baseline for such further research by identifying important traits in individual bacterial strains that are associated with enhancing or inhibiting cell growth, as well as important host factors that determine sensitivity to those bacterial traits. The influence of individual bacterial traits should be the focus of follow-up research and can be investigated with add-back experiments, by introducing, e.g. virulence genes in non-virulent strains.

## Conclusions

Above, we presented the results of the first large-scale analysis of the effects of gut bacteria on CRC cell growth. Our results with bacterial cells and secretomes reflect the complexity of the microbe–host interactions in the human gut. First, bacterial families tend to have consistent effects on cell growth, although these effects are not universal. This may partially be explained by the presence of virulence genes in some strains. Second, there are cell line dependent effects in cells due to their mutational landscape heterogeneity. Notably, these cell lines have acquired several different cancer hallmarks, yet their growth can still be influenced by specific bacteria. Our results suggest that the response of epithelial cells to gut bacteria may differ between patients due to differences in their gut bacteria. The relevance of our *in vitro* results for patients and CRC specificity remains to be studied. Cell growth enhancing and inhibiting bacterial traits could be important for determining risk for cancer or its progression, and these consequences could potentially be counteracted by microbiome modulation. Currently, microbiome medicine applications have already been adapted to some other diseases. For instance, *Akkermansia muciniphila* was demonstrated to improve diabetes type 2 and obesity in mice.^17,18^ Resistant life-threatening *Clostridium difficile* infections and ulcerative colitis were successfully treated with fecal microbiota transplants (FMT).^19,20^ Our study highlights the complex processes at play at the gut–microbiome interface and contributes to a better understanding of the role of different bacterial strains in altering colonic epithelial cell proliferation.

## Supplementary Material

Supplemental Material

## References

[1] Sender R, Fuchs S, Milo R. Revised estimates for the number of human and bacteria cells in the body. PLoS Biol. 2016;14:e1002533–e. doi:10.1371/journal.pbio.1002533. 27541692 PMC4991899

[2] Rajilić-Stojanović M, de Vos WM. The first 1000 cultured species of the human gastrointestinal microbiota. FEMS Microbiol Rev. 2014;38:996–1047. doi:10.1111/1574-6976.12075. 24861948 PMC4262072

[3] Qin J, Li R, Raes J, Arumugam M, Burgdorf KS, Manichanh C, Nielsen T, Pons N, Levenez F, Yamada T, et al. A human gut microbial gene catalogue established by metagenomic sequencing. Nature. 2010;464:59–65. doi:10.1038/nature08821. 20203603 PMC3779803

[4] Arnold M, Sierra MS, Laversanne M, Soerjomataram I, Jemal A, Bray F. Global patterns and trends in colorectal cancer incidence and mortality. Gut. 2017;66:683–691. doi:10.1136/gutjnl-2015-310912. 26818619

[5] Gao R, Kong C, Huang L, Li H, Qu X, Liu Z, Lan P, Wang J, Qin H. Mucosa-associated microbiota signature in colorectal cancer. Eur J Clin Microbiol Infect Dis Off Publ Eur Soc Clin Microbiol. 2017;36:2073–2083. doi:10.1007/s10096-017-3026-4. 28600626

[6] Tjalsma H, Boleij A, Marchesi JR, Dutilh BE. A bacterial driver-passenger model for colorectal cancer: beyond the usual suspects. Nat Rev Microbiol. 2012;10:575–582. doi:10.1038/nrmicro2819. 22728587

[7] Gao Z, Guo B, Gao R, Zhu Q, Qin H. Microbiota disbiosis is associated with colorectal cancer. Front Microbiol. 2015;6:20. doi:10.3389/fmicb.2015.00020. 25699023 PMC4313696

[8] Hanahan D, Weinberg RA. Hallmarks of cancer: the next generation. Cell. 2011;144:646–674. doi:10.1016/j.cell.2011.02.013. 21376230

[9] Marchesi JR, Dutilh BE, Hall N, Peters WHM, Roelofs R, Boleij A, Tjalsma H. Towards the human colorectal cancer microbiome. PloS One. 2011;6:e20447–e. doi:10.1371/journal.pone.0020447. 21647227 PMC3101260

[10] Wirbel J, Pyl PT, Kartal E, Zych K, Kashani A, Milanese A, Fleck JS, Voigt AY, Palleja A, Ponnudurai R, et al. Meta-analysis of fecal metagenomes reveals global microbial signatures that are specific for colorectal cancer. Nat Med. 2019;25:679–689. doi:10.1038/s41591-019-0406-6. 30936547 PMC7984229

[11] Xu K, Jiang B. Analysis of Mucosa-associated microbiota in colorectal cancer. Med Sci Monit Int Med J Exp Clin Res. 2017;23:4422–4430. doi:10.12659/MSM.904220. PMC5609654 28904330

[12] Donia MS. A toolbox for microbiome engineering. Cell Syst. 2015;1:21–23. doi:10.1016/j.cels.2015.07.003. 27135687

[13] Foo JL, Ling H, Lee YS, Chang MW. Microbiome engineering: current applications and its future. Biotechnol J. 2017;12. doi:10.1002/biot.201600099. 10.1002/biot.201600099 28133942

[14] Garcia-Jimenez B, de la Rosa T, Wilkinson MD. MDPbiome: microbiome engineering through prescriptive perturbations. Bioinformatics (Oxford, England). 2018;34:i838–i47. doi:10.1093/bioinformatics/bty562. PMC6129268 30423107

[15] Sonnenburg JL. Microbiome engineering. Nature. 2015;518:S10. doi:10.1038/518S10a. 25715274

[16] Bernardes N, Seruca R, Chakrabarty AM, Fialho AM. Microbial-based therapy of cancer: current progress and future prospects. Bioeng Bugs. 2010;1:178–190. doi:10.4161/bbug.1.3.10903. 21326924 PMC3026423

[17] Song S, Vuai MS, Zhong M. The role of bacteria in cancer therapy - enemies in the past, but allies at present. Infect Agent Cancer. 2018;13:9. doi:10.1186/s13027-018-0180-y. 29568324 PMC5856380

[18] Lukasiewicz K, Fol M. Microorganisms in the treatment of cancer: advantages and limitations. J Immunol Res. 2018;2018:2397808. doi:10.1155/2018/2397808. 29682586 PMC5848056

[19] Forbes NS, Coffin RS, Deng L, Evgin L, Fiering S, Giacalone M, Gravekamp C, Gulley JL, Gunn H, Hoffman RM, et al. White paper on microbial anti-cancer therapy and prevention. J ImmunoTher Cancer. 2018;6:78. doi:10.1186/s40425-018-0381-3. 30081947 PMC6091193

[20] Kramer MG, Masner M, Ferreira FA, Hoffman RM. Bacterial therapy of cancer: promises, limitations, and insights for future directions. Front Microbiol. 2018;9:16. doi:10.3389/fmicb.2018.00016. 29472896 PMC5810261

[21] Shen XJ, Rawls JF, Randall T, Burcal L, Mpande CN, Jenkins N, Jovov B, Abdo Z, Sandler RS, Keku TO. Molecular characterization of mucosal adherent bacteria and associations with colorectal adenomas. Gut Microbes. 2010;1:138–147. doi:10.4161/gmic.1.3.12360. 20740058 PMC2927011

[22] Chen W, Liu F, Ling Z, Tong X, Xiang C. Human intestinal lumen and mucosa-associated microbiota in patients with colorectal cancer. PloS One. 2012;7:e39743. doi:10.1371/journal.pone.0039743. 22761885 PMC3386193

[23] Flemer B, Lynch DB, Brown JM, Jeffery IB, Ryan FJ, Claesson MJ, O'Riordain M, Shanahan F, O'Toole PW. Tumour-associated and non-tumour-associated microbiota in colorectal cancer. Gut. 2017;66:633–643. doi:10.1136/gutjnl-2015-309595. 26992426 PMC5529966

[24] Zeller G, Tap J, Voigt AY, Sunagawa S, Kultima JR, Costea PI, Amiot A, Bohm J, Brunetti F, Habermann N, et al. Potential of fecal microbiota for early-stage detection of colorectal cancer. Mol Syst Biol. 2014;10:766. doi:10.15252/msb.20145645. 25432777 PMC4299606

[25] Wang T, Cai G, Qiu Y, Fei N, Zhang M, Pang X, Jia W, Cai S, Zhao L. Structural segregation of gut microbiota between colorectal cancer patients and healthy volunteers. Isme J. 2012;6:320–329. doi:10.1038/ismej.2011.109. 21850056 PMC3260502

[26] Hale VL, Jeraldo P, Mundy M, Yao J, Keeney G, Scott N, Cheek EH, Davidson J, Greene M, Martinez C, et al. Synthesis of multi-omic data and community metabolic models reveals insights into the role of hydrogen sulfide in colon cancer. Methods (San Diego, Calif). 2018;149:59–68. doi:10.1016/j.ymeth.2018.04.024. PMC6191348 29704665

[27] Boleij A, Hechenbleikner EM, Goodwin AC, Badani R, Stein EM, Lazarev MG, Ellis B, Carroll KC, Albesiano E, Wick EC, et al. The Bacteroides fragilis toxin gene is prevalent in the colon mucosa of colorectal cancer patients. Clin Infect Dis Offl Publ Infect Dis Soc Am. 2015;60:208–215. doi:10.1093/cid/ciu787. PMC4351371 25305284

[28] Kostic AD, Gevers D, Pedamallu CS, Michaud M, Duke F, Earl AM, Ojesina AI, Jung J, Bass AJ, Tabernero J, et al. Genomic analysis identifies association of Fusobacterium with colorectal carcinoma. Genome Res. 2012;22:292–298. doi:10.1101/gr.126573.111. 22009990 PMC3266036

[29] Boleij A, Dutilh BE, Kortman GA, Roelofs R, Laarakkers CM, Engelke UF, Tjalsma H. Bacterial responses to a simulated colon tumor microenvironment. MCP. 2012;11:851–862. doi:10.1074/mcp.M112.019315. 22713208 PMC3494137

[30] Tjalsma H, Bolhuis A, Jongbloed JD, Bron S, van Dijl JM. Signal peptide-dependent protein transport in Bacillus subtilis: a genome-based survey of the secretome. MMBR. 2000;64:515–547. 10974125 10.1128/mmbr.64.3.515-547.2000 PMC99003

[31] Rubinstein MR, Wang X, Liu W, Hao Y, Cai G, Han YW. Fusobacterium nucleatum promotes colorectal carcinogenesis by modulating E-cadherin/beta-catenin signaling via its FadA adhesin. Cell Host Microbe. 2013;14:195–206. doi:10.1016/j.chom.2013.07.012. 23954158 PMC3770529

[32] Fardini Y, Wang X, Temoin S, Nithianantham S, Lee D, Shoham M, Han YW. Fusobacterium nucleatum adhesin FadA binds vascular endothelial cadherin and alters endothelial integrity. Mol Microbiol. 2011;82:1468–1480. doi:10.1111/j.1365-2958.2011.07905.x. 22040113 PMC3237733

[33] Chen Y, Peng Y, Yu J, Chen T, Wu Y, Shi L, Li Q, Wu J, Fu X. Invasive Fusobacterium nucleatum activates beta-catenin signaling in colorectal cancer via a TLR4/P-PAK1 cascade. Oncotarget. 2017;8:31802–31814. doi:10.18632/oncotarget.15992. 28423670 PMC5458249

[34] Donnenberg MS, Tacket CO, James SP, Losonsky G, Nataro JP, Wasserman SS, Kaper JB, Levine MM. Role of the eaeA gene in experimental enteropathogenic Escherichia coli infection. J Clin Invest. 1993;92:1412–1417. doi:10.1172/JCI116717. 8376594 PMC288285

[35] Jerse AE, Yu J, Tall BD, Kaper JB. A genetic locus of enteropathogenic Escherichia coli necessary for the production of attaching and effacing lesions on tissue culture cells. Proc Natl Acad Sci U S A. 1990;87:7839–7843. doi:10.1073/pnas.87.20.7839. 2172966 PMC54845

[36] Maddocks OD, Short AJ, Donnenberg MS, Bader S, Harrison DJ. Attaching and effacing Escherichia coli downregulate DNA mismatch repair protein in vitro and are associated with colorectal adenocarcinomas in humans. PloS One. 2009;4:e5517. doi:10.1371/journal.pone.0005517. 19436735 PMC2677459

[37] Rhee KJ, Wu S, Wu X, Huso DL, Karim B, Franco AA, Rabizadeh S, Golub JE, Mathews LE, Shin J, et al. Induction of persistent colitis by a human commensal, enterotoxigenic Bacteroides fragilis, in wild-type C57BL/6 mice. Infect Immun. 2009;77:1708–1718. doi:10.1128/IAI.00814-08. 19188353 PMC2663167

[38] Wu S, Morin PJ, Maouyo D, Sears CL. Bacteroides fragilis enterotoxin induces c-Myc expression and cellular proliferation. Gastroenterology. 2003;124:392–400. doi:10.1053/gast.2003.50047. 12557145

[39] Cuevas-Ramos G, Petit CR, Marcq I, Boury M, Oswald E, Nougayrede JP. Escherichia coli induces DNA damage in vivo and triggers genomic instability in mammalian cells. Proc Natl Acad Sci U S A. 2010;107:11537–11542. doi:10.1073/pnas.1001261107. 20534522 PMC2895108

[40] Arthur JC, Perez-Chanona E, Muhlbauer M, Tomkovich S, Uronis JM, Fan TJ, Campbell BJ, Abujamel T, Dogan B, Rogers AB, et al. Intestinal inflammation targets cancer-inducing activity of the microbiota. Science (New York, NY). 2012;338:120–123. doi:10.1126/science.1224820. PMC3645302 22903521

[41] Wilson MR, Jiang Y, Villalta PW, Stornetta A, Boudreau PD, Carra A, Brennan CA, Chun E, Ngo L, Samson LD, et al. The human gut bacterial genotoxin colibactin alkylates DNA. Science (New York, NY). 2019;363. 10.1126/science.aar7785 PMC6407708 30765538

[42] Wattam AR, Davis JJ, Assaf R, Boisvert S, Brettin T, Bun C, Conrad N, Dietrich EM, Disz T, Gabbard JL, et al. Improvements to PATRIC, the all-bacterial bioinformatics database and analysis resource center. Nucleic Acids Res. 2017;45:D535–d42. doi:10.1093/nar/gkw1017. 27899627 PMC5210524

[43] Boleij A, van Gelder MM, Swinkels DW, Tjalsma H. Clinical Importance of Streptococcus gallolyticus infection among colorectal cancer patients: systematic review and meta-analysis. Clin Infect Dis Offl Publ Infect Dis Soc Am. 2011;53:870–878. doi:10.1093/cid/cir609. 21960713

[44] Wentling GK, Metzger PP, Dozois EJ, Chua HK, Krishna M. Unusual bacterial infections and colorectal carcinoma–Streptococcus bovis and Clostridium septicum: report of three cases. Dis Colon Rectum. 2006;49:1223–1227. doi:10.1007/s10350-006-0576-4. 16845563

[45] Schaaf RE, Jacobs N, Kelvin FM, Gallis HA, Akwari O, Thompson WM. Clostridium septicum infection associated with colonic carcinoma and hematologic abnormality. Radiology. 1980;137:625–627. doi:10.1148/radiology.137.3.6934562. 6934562

[46] Sidhu JS, Mandal A, Virk J, Gayam V. Early detection of colon cancer following incidental finding of Clostridium septicum bacteremia. J Invest Med High Impact Case Rep. 2019;7:2324709619832050. doi:10.1177/2324709619832050. PMC6415464 30857430

[47] Zheng Y, Luo Y, Lv Y, Huang C, Sheng Q, Zhao P, Ye J, Jiang W, Liu L, Song X, et al. Clostridium difficile colonization in preoperative colorectal cancer patients. Oncotarget. 2017;8:11877–11886. doi:10.18632/oncotarget.14424. 28060753 PMC5355311

[48] Fukugaiti MH, Ignacio A, Fernandes MR, Ribeiro Junior U, Nakano V, Avila-Campos MJ. High occurrence of Fusobacterium nucleatum and Clostridium difficile in the intestinal microbiota of colorectal carcinoma patients. Braz J Microbiol [Publ Braz Soc Microbiol]. 2015;46:1135–1140. doi:10.1590/S1517-838246420140665. PMC4704648 26691472

[49] Yamazaki K, Tsunoda A, Sibusawa M, Tsunoda Y, Kusano M, Fukuchi K, Yamanaka M, Kushima M, Nomoto K, Morotomi M. The effect of an oral administration of Lactobacillus casei strain shirota on azoxymethane-induced colonic aberrant crypt foci and colon cancer in the rat. Oncol Rep. 2000;7:977–982. doi:10.3892/or.7.5.977. 10948325

[50] Kato-Kataoka A, Nishida K, Takada M, Kawai M, Kikuchi-Hayakawa H, Suda K, Ishikawa H, Gondo Y, Shimizu K, Matsuki T, et al. Fermented milk containing lactobacillus casei strain shirota preserves the diversity of the gut microbiota and relieves abdominal dysfunction in healthy medical students exposed to academic stress. Appl Environ Microbiol. 2016;82:3649–3658. doi:10.1128/AEM.04134-15. 27208120 PMC4959178

[51] Morishita T, Fukada T, Shirota M, Yura T. Genetic basis of nutritional requirements in Lactobacillus casei. J Bacteriol. 1974;120:1078–1084. doi:10.1128/JB.120.3.1078-1084.1974. 4215789 PMC245885

[52] Derrien M, Vaughan EE, Plugge CM, de Vos WM. Akkermansia muciniphila gen. nov., sp. nov., a human intestinal mucin-degrading bacterium. Int J Syst Evol Microbiol. 2004;54:1469–1476. doi:10.1099/ijs.0.02873-0. 15388697

[53] Dejea CM, Fathi P, Craig JM, Boleij A, Taddese R, Geis AL, Wu X, DeStefano Shields CE, Hechenbleikner EM, Huso DL, et al. Patients with familial adenomatous polyposis harbor colonic biofilms containing tumorigenic bacteria. Science (New York, NY). 2018;359:592–597. doi:10.1126/science.aah3648. PMC5881113 29420293

[54] Weir TL, Manter DK, Sheflin AM, Barnett BA, Heuberger AL, Ryan EP. Stool microbiome and metabolome differences between colorectal cancer patients and healthy adults. PloS One. 2013;8:e70803. doi:10.1371/journal.pone.0070803. 23940645 PMC3735522

[55] Dingemanse C, Belzer C, van Hijum SA, Gunthel M, Salvatori D, den Dunnen JT, Kuijper EJ, Devilee P, de Vos WM, van Ommen GB, et al. Akkermansia muciniphila and Helicobacter typhlonius modulate intestinal tumor development in mice. Carcinogenesis. 2015;36:1388–1396. doi:10.1093/carcin/bgv120. 26320104

[56] Knapp O, Maier E, Mkaddem SB, Benz R, Bens M, Chenal A, Geny B, Vandewalle A, Popoff MR. Clostridium septicum alpha-toxin forms pores and induces rapid cell necrosis. Toxicon. 2010;55:61–72. doi:10.1016/j.toxicon.2009.06.037. 19632260

[57] Wust J, Hardegger U. Transferable resistance to clindamycin, erythromycin, and tetracycline in Clostridium difficile. Antimicrob Agents Chemother. 1983;23:784–786. doi:10.1128/AAC.23.5.784. 6870225 PMC184818

[58] Boleij A, Muytjens CM, Bukhari SI, Cayet N, Glaser P, Hermans PW, Swinkels DW, Bolhuis A, Tjalsma H.Novel clues on the specific association of Streptococcus gallolyticus subsp gallolyticus with colorectal cancer. J Infect Dis. 2011;203:1101–1109. doi:10.1093/infdis/jiq169. 21451000

[59] Tripodi MF, Fortunato R, Utili R, Triassi M, Zarrilli R. Molecular epidemiology of Streptococcus bovis causing endocarditis and bacteraemia in Italian patients. Clin Microbiol Infect Off Publ Eur Soc Clin Microbiol Infect Dis. 2005;11:814–819. doi:10.1111/j.1469-0691.2005.01248.x. 16153255

[60] Tripodi MF, Adinolfi LE, Ragone E, Durante Mangoni E, Fortunato R, Iarussi D, Ruggiero G, Utili R. Streptococcus bovis endocarditis and its association with chronic liver disease: an underestimated risk factor. Clin Infect Dis Offl Publ Infect Dis Soc Am. 2004;38:1394–1400. doi:10.1086/392503. 15156477

[61] Ogawa Y, Ooka T, Shi F, Ogura Y, Nakayama K, Hayashi T, Shimoji Y. The genome of Erysipelothrix rhusiopathiae, the causative agent of swine erysipelas, reveals new insights into the evolution of firmicutes and the organism’s intracellular adaptations. J Bacteriol. 2011;193:2959–2971. doi:10.1128/JB.01500-10. 21478354 PMC3133210

[62] Taddese R, Belzer C, Aalvink S, de Jonge MI, Nagtegaal ID, Dutilh BE, Boleij A. Bacterial zombies and ghosts: production of inactivated gram-positive and gram-negative species with preserved cellular morphology and cytoplasmic content. bioRxiv. 2018;458158. 10.1016/j.mimet.2021.106208 33766606

[63] Ahmed D, Eide PW, Eilertsen IA, Danielsen SA, Eknaes M, Hektoen M, Lind GE, Lothe RA. Epigenetic and genetic features of 24 colon cancer cell lines. Oncogenesis. 2013;2:e71. doi:10.1038/oncsis.2013.35. 24042735 PMC3816225

[64] Eijkelenboom A, Kamping EJ, Kastner-van Raaij AW, Hendriks-Cornelissen SJ, Neveling K, Kuiper RP, Hoischen A, Nelen MR, Ligtenberg MJ, Tops BB. Reliable next-generation sequencing of formalin-fixed, paraffin-embedded tissue using single molecule tags. JMD. 2016;18:851–863. doi:10.1016/j.jmoldx.2016.06.010. 27637301

[65] Mosmann T. Rapid colorimetric assay for cellular growth and survival: application to proliferation and cytotoxicity assays. J Immunol Methods. 1983;65:55–63. doi:10.1016/0022-1759(83)90303-4. 6606682

[66] van der Maaten LH, Geoffrey. Visualizing data using t-SNE. J Mach Learn Res. 2008;9:2579–2605.

[67] Bentley JL. Multidimensional binary search trees used for associative searching. Commun ACM. 1975;18:509–517.

[68] Ballard J, Sokolov Y, WL Y, BL K, Tweten RK. Activation and mechanism of Clostridium septicum alpha toxin. Mol Microbiol. 1993;10:627–634. 7968539 10.1111/j.1365-2958.1993.tb00934.x

[69] Aziz RK, Bartels D, Best AA, DeJongh M, Disz T, Edwards RA, Formsma K, Gerdes S, Glass EM, Kubal M, et al. The RAST Server: rapid annotations using subsystems technology. BMC Genomics. 2008;9:75. doi:10.1186/1471-2164-9-75. 18261238 PMC2265698

[70] Poisson Distribution. Univariate Discrete Distributions. 2005;156–207.

[71] Graham FL, Smiley J, Russell WC, Nairn R. Characteristics of a human cell line transformed by DNA from human adenovirus type 5. J Gen Virol. 1977;36:59–74. doi:10.1099/0022-1317-36-1-59. 886304

[72] Ma CT, Luo HS, Gao F, Tang QC, Chen W. Fusobacterium nucleatum promotes the progression of colorectal cancer by interacting with E-cadherin. Oncol Lett. 2018;16:2606–2612. doi:10.3892/ol.2018.8947. 30013655 PMC6036566

[73] Dalmasso G, Cougnoux A, Delmas J, Darfeuille-Michaud A, Bonnet R. The bacterial genotoxin colibactin promotes colon tumor growth by modifying the tumor microenvironment. Gut Microbes. 2014;5:675–680. doi:10.4161/19490976.2014.969989. 25483338 PMC4615906

[74] Fais T, Delmas J, Barnich N, Bonnet R, Dalmasso G. Colibactin: more than a new bacterial toxin. Toxins. 2018;10. doi:10.3390/toxins10040151. 10.3390/toxins10040151 PMC5923317 29642622

[75] Dean P, Kenny B. The effector repertoire of enteropathogenic E. coli: ganging up on the host cell. Curr Opin Microbiol. 2009;12:101–109. doi:10.1016/j.mib.2008.11.006. 19144561 PMC2697328

[76] Singh AP, Sharma S, Pagarware K, Siraji RA, Ansari I, Mandal A, Walling P, Aijaz S. Enteropathogenic E. coli effectors EspF and Map independently disrupt tight junctions through distinct mechanisms involving transcriptional and post-transcriptional regulation. Sci Rep. 2018;8:3719. doi:10.1038/s41598-018-22017-1. 29487356 PMC5829253

[77] Wassenaar TM. E. coli and colorectal cancer: a complex relationship that deserves a critical mindset. Crit Rev Microbiol. 2018;44:619–632. doi:10.1080/1040841X.2018.1481013. 29909724

[78] Bossuet-Greif N, Vignard J, Taieb F, Mirey G, Dubois D, Petit C, Oswald E, Nougayrede JP. The colibactin genotoxin generates DNA Interstrand cross-links in infected cells. mBio. 2018;9. 10.1128/mBio.02393-17 PMC5874909 29559578

[79] Ahn J, Sinha R, Pei Z, Dominianni C, Wu J, Shi J, Goedert JJ, Hayes RB, Yang L. Human gut microbiome and risk for colorectal cancer. J Natl Cancer Inst. 2013;105:1907–1911. doi:10.1093/jnci/djt300. 24316595 PMC3866154

[80] Yang Y, Weng W, Peng J, Hong L, Yang L, Toiyama Y, Gao R, Liu M, Yin M, Pan C, et al. Fusobacterium nucleatum increases proliferation of colorectal cancer cells and tumor development in mice by activating toll-like receptor 4 signaling to nuclear factor-kappaB, and up-regulating expression of MicroRNA-21. Gastroenterology. 2017;152:851–66.e24. doi:10.1053/j.gastro.2016.11.018. 27876571 PMC5555435

[81] Castellarin M, Warren RL, Freeman JD, Dreolini L, Krzywinski M, Strauss J, Barnes R, Watson P, Allen-Vercoe E, Moore RA, et al. Fusobacterium nucleatum infection is prevalent in human colorectal carcinoma. Genome Res. 2012;22:299–306. doi:10.1101/gr.126516.111. 22009989 PMC3266037

[82] Liu J, Hsieh CL, Gelincik O, Devolder B, Sei S, Zhang S, Lipkin SM, Chang YF. Proteomic characterization of outer membrane vesicles from gut mucosa-derived fusobacterium nucleatum. J Proteomics. 2019;195:125–137. doi:10.1016/j.jprot.2018.12.029. 30634002

[83] Han YW, Ikegami A, Rajanna C, Kawsar HI, Zhou Y, Li M, Sojar HT, Genco RJ, Kuramitsu HK, Deng CX. Identification and characterization of a novel adhesin unique to oral fusobacteria. J Bacteriol. 2005;187:5330–5340. doi:10.1128/JB.187.15.5330-5340.2005. 16030227 PMC1196005

[84] Sears CL. The toxins of Bacteroides fragilis. Toxicon. 2001;39:1737–1746. doi:10.1016/S0041-0101(01)00160-X. 11595636

[85] Jans C, Boleij A. The road to infection: host-microbe interactions defining the pathogenicity of Streptococcus bovis/Streptococcus equinus complex members. Front Microbiol. 2018;9:603. doi:10.3389/fmicb.2018.00603. 29692760 PMC5902542

[86] Boleij A, Tjalsma H. The itinerary of Streptococcus gallolyticus infection in patients with colonic malignant disease. Lancet Infect Dis. 2013;13:719–724. doi:10.1016/S1473-3099(13)70107-5. 23831427

[87] Kumar R, Herold JL, Schady D, Davis J, Kopetz S, Martinez-Moczygemba M, Murray BE, Han F, Li Y, Callaway E, et al. Streptococcus gallolyticus subsp. gallolyticus promotes colorectal tumor development. PLoS Pathog. 2017;13:e1006440. doi:10.1371/journal.ppat.1006440. 28704539 PMC5509344

[88] Yang J, Zhang W, Evans PM, Chen X, He X, Liu C. Adenomatous polyposis coli (APC) differentially regulates beta-catenin phosphorylation and ubiquitination in colon cancer cells. J Biol Chem. 2006;281:17751–17757. doi:10.1074/jbc.M600831200. 16798748

[89] Tate JG, Bamford S, Jubb HC, Sondka Z, Beare DM, Bindal N, Boutselakis H, Cole CG, Creatore C, Dawson E, et al. COSMIC: the Catalogue Of Somatic Mutations In Cancer. Nucleic Acids Res. 2019;47:D941–d7. doi:10.1093/nar/gky1015. 30371878 PMC6323903

[90] Sgro GG, Oka GU, Souza DP, Cenens W, Bayer-Santos E, Matsuyama BY, Bueno NF, Dos Santos TR, Alvarez-Martinez CE, Salinas RK, et al. Bacteria-killing Type IV secretion systems. Front Microbiol. 2019;10:1078. doi:10.3389/fmicb.2019.01078. 31164878 PMC6536674

[91] Kiersztyn B, Siuda W, Chrost RJ. Persistence of bacterial proteolytic enzymes in lake ecosystems. FEMS Microbiol Ecol. 2012;80:124–134. doi:10.1111/j.1574-6941.2011.01276.x. 22150269

[92] Battaglia-Hsu SF, Akchiche N, Noel N, Alberto JM, Jeannesson E, Orozco-Barrios CE, Martinez-Fong D, Daval JL, Gueant JL. Vitamin B12 deficiency reduces proliferation and promotes differentiation of neuroblastoma cells and up-regulates PP2A, proNGF, and TACE. Proc Natl Acad Sci U S A. 2009;106:21930–21935. doi:10.1073/pnas.0811794106. 19959661 PMC2788478

[93] Wexler AG, Schofield WB, Degnan PH, Folta-Stogniew E, Barry NA, Goodman AL. Human gut Bacteroides capture vitamin B12 via cell surface-exposed lipoproteins. Elife. 2018;7. doi:0.7554/eLife.37138. 10.7554/eLife.37138 PMC6143338 30226189

[94] Patel D, Witt SN. Ethanolamine and Phosphatidylethanolamine: partners in Health and Disease. Oxid Med Cell Longev. 2017;2017:4829180. doi:10.1155/2017/4829180. 28785375 PMC5529665

[95] Tsoy O, Ravcheev D, Mushegian A. Comparative genomics of ethanolamine utilization. J Bacteriol. 2009;191:7157–7164. doi:10.1128/JB.00838-09. 19783625 PMC2786565

[96] Kaval KG, Garsin DA. Ethanolamine utilization in bacteria. mBio. 2018;9. doi:0.1128/mBio.00066-18. 10.1128/mBio.00066-18 PMC5821096 29463652

[97] Kendall MM, Gruber CC, Parker CT, Sperandio V. Ethanolamine controls expression of genes encoding components involved in interkingdom signaling and virulence in enterohemorrhagic Escherichia coli O157:H7. mBio. 2012;3. doi:10.1128/mBio.00050-12. PMC3372972 22589288

[98] van de Wetering M, Francies HE, Francis JM, Bounova G, Iorio F, Pronk A, van Houdt W, van Gorp J, Taylor-Weiner A, Kester L, et al. Prospective derivation of a living organoid biobank of colorectal cancer patients. Cell. 2015;161:933–945. doi:10.1016/j.cell.2015.03.053. 25957691 PMC6428276

