# Supplementary material for: Growth rate alterations of human colorectal cancer cells by 157 gut bacteria
Source: Gut Microbes. 2020 Sep 11;12(1):1799733. doi: 10.1080/19490976.2020.1799733 (PMC7524400; doi:10.1080/19490976.2020.1799733)
Supplement: Supplemental Material [file KGMI_A_1799733_SM4517.zip › Supplementary information/Supplementary Figure captions.docx]

**Supplementary Figure captions**

**S1. Average growth of human cell lines exposed to bacterial cells and secretomes.** Colored lines and error bars show the average and standard deviation of MTT measurements for 6 human cell lines exposed to cells and secretomes of 145 and 154 bacteria, respectively. Measurements were recorded at the incubation times of 0, 24, 48, and 72h. The dark lines show the average of the cell line without bacterial products (negative controls).

**S2. Reproducibility of four experimental replicates.** Scatter plots of the pairwise combinations of four experimental replicates of MTT measurements at incubation times of 0, 24, 48, and 72h. Individual plots exhibit measurements of all possible combinations of two of the four experimental replicates of one cell line exposed to the cells or secretomes of, respectively 145 and 154 bacterial strains. Experimental replicates were concatenated in the x and y variables in such a way that all pairwise comparisons were made (replicate 1 vs. 2, 1 vs. 3, 1 vs. 4, 2 vs. 3, 2 vs. 4, and 3 vs. 4), i.e. variable x has replicate 1, 1, 2, 2,and 3, while y contains replicate 2, 3, 4, 3 and 4. Lines show the best fit to a linear model. The specific linear regression coefficients, intercepts, R2, and p-values are shown on the top of each plot.

**S3. Cell growth rate alterations ordered by phylogenomic distances.** Heatmaps show the growth rate alterations of six cell lines incubated with the cells and secretomes of strains from seven bacterial families. Rows of the heatmap are ranked according to the tips of the phylogenomic trees shown on the left.

**S4. *Streptococcus* phylogenomic tree.** Genome-based phylogenetic reconstruction of 238 Streptococcus genomes confirms that the genome sequences of the *S. bovis* strains reported in this study are closely related to previously sequenced genomes of *S. gallolyticus* and *S. pasteurianus*. The taxa names of strains used in our study are highlighted red. Monophyletic branches containing strains from a single species are collapsed and highlighted with blue triangles.

**S5. Linear relation between cell counts and MTT measurements.**

Cell growth was recorded by two different methods (MTT assay and cell counting) and plotted on a linear x-y graph. Both methods were significantly correlated (p<0.0001, Pearson r=0.813).

**Supplementary Table captions**

**S1. Bacterial strains used in this study.**

**S2. Cancer mutational profile of six human cell lines used in this study.**

**S3. Growth rate scores, z-scores, and p-values measured from human cells incubated with bacterial cells. These values were computed from the average of four experimental replicates (see “cell growth analysis” in the methods section).**

**S4. Growth rate scores, z-scores, and p-values measured from human cells incubated with bacterial secretomes. These values were computed from the average of four experimental replicates (see “cell growth analysis” in the methods section)**

**S5. Literature summary of microbial virulence factors potentially associated to cancer.**

**S6. Correlation between the effects of bacterial cells and secretomes on growth rate.** The correlation values were obtained from the growth rate scores computed from the average of four experimental replicates of bacterial cells and secretomes for the group of strains that belong to the indicated bacterial family.

**S7. Statistical significance analysis of family-specific clustering of the growth rate scores.**

**S8. Correlation between the pairwise phylogenetic distance between bacterial strains used in this study and the pairwise Euclidean distance of the growth rate scores.**

**S9. Distribution of genes coding for virulence factors in the genomes of the bacteria used in this study.** Toxins of *Clostridia* were present in bacteria of the *Clostridiales* order while other toxins were present within bacterial families.

**S10. Functional genomic terms significantly associated to growth rate scores within bacterial families.**
