# Supplementary material for: Growth rate alterations of human colorectal cancer cells by 157 gut bacteria
Source: Gut Microbes. 2020 Sep 11;12(1):1799733. doi: 10.1080/19490976.2020.1799733 (PMC7524400; doi:10.1080/19490976.2020.1799733)

Reproducibility of experimental replicates

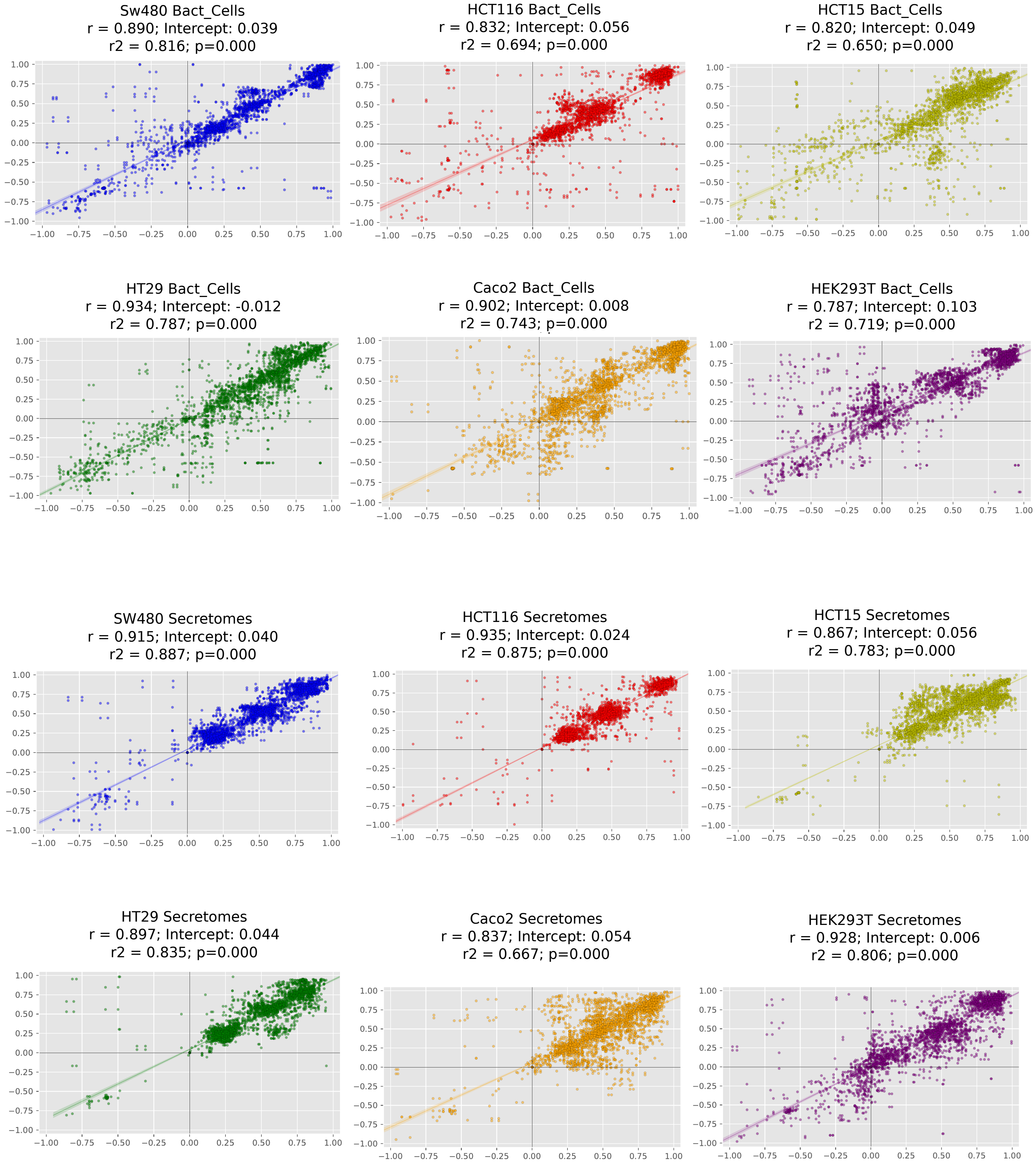

Supplement: Supplemental Material [file KGMI_A_1799733_SM4517.zip › Supplementary information/Supplementary_Figure_S2.pdf]
